# Juvenile idiopathic arthritis classification

**DOI:** 10.1186/1546-0096-12-S1-I3

**Published:** 2014-09-17

**Authors:** Alberto Martini

**Affiliations:** 1 Department of Pediatrics, University of Genoa; 2 Pediatria II e Reumatologia, Istituto G Gaslini, Genoa, Italy

## 

Juvenile idiopathic arthritis (JIA) is not a disease, but an exclusion diagnosis that encompasses all forms of arthritis that begin before the age of 16 years, persist for more than 6 weeks, and are of unknown origin. This heterogeneous group of chronic arthritides has been classified on clinical and laboratory grounds to try to identify homogeneous, mutually exclusives categories suitable for etiopathogenic studies. During the last years evidence has accumulated suggesting that while some JIA categories identify quite definite disease entities, others represent heterogeneous conditions and in particular that a homogeneous disease entity (ANA positive, early onset oligoarthritis) is included in several different JIA categories. These and other findings suggest the need to reconsider some aspects of the current International League of Associations for Rheumatology (ILAR) JIA classification and nomenclature.

## Disclosure of interest

None declared.

